# The medical consequences of eating disorders: the correlation between the severity of the disease and the degree of the cardiological changes in paediatric patients with anorexia nervosa

**DOI:** 10.1192/j.eurpsy.2023.905

**Published:** 2023-07-19

**Authors:** E. Adamo, T. Pisano

**Affiliations:** University of Florence, Firenze, Italy

## Abstract

**Introduction:**

Anorexia nervosa (AN) is associated with several medical complications. The cardiac changes represent the most severe complications and are associated with higher mortality. For this reason, periodic evaluation is necessary, by ECG and echocardiography. Moreover, there is not a protocol that defines the timelines or how to select higher risk patients that must be evaluated more frequently.

**Objectives:**

This single-center, retrospective, observational and epidemiological study aims to analyze the prevalence of cardiac changes and their correlation with disease severity, in patients admitted to the Child and Adolescent Psychiatry Unit, AOU Meyer, Florence.

**Methods:**

The study population consisted of 123 children between the ages of 7 and 18 years old admitted to inpatient or intensive day hospital, with a diagnosis of AN, between January 1, 2019, to March 31, 2022. Data were collected by retrospectively consulting clinical reports. The correlation between BMI, percentage of weight loss since the onset of symptoms and HR, QTc interval values and pericardial effusion was evaluated. Furthermore, the correlation between cardiac changes and the intake of antidepressant and antipsychotic medications was analyzed.

**Results:**

The overall prevalence of cardiac changes was 57.7%. In our analysis BMI showed a significant positive correlation whit HR. QTc prolongation was significantly related only to psychotropic drug intake. Pericardial effusion was evidenced exclusively in patients diagnosed with severe or extreme AN. After six months from the hospitalization and the beginning of treatment the prevalence of cardiovascular complications was reduced.

**Conclusions:**

The present study identifies criteria able to select patients with AN at higher risk of developing cardiac changes and underlines the importance of performing more frequent and targeted cardiologic evaluations in this subgroup of patients. This suggests the importance of establishing a common protocol for all clinicians.

**Disclosure of Interest:**

None Declared

